# Abnormal resting-state functional connectivity of hippocampal subregions in children with primary nocturnal enuresis

**DOI:** 10.3389/fpsyt.2022.966362

**Published:** 2022-08-22

**Authors:** Shaogen Zhong, Lichi Zhang, Mengxing Wang, Jiayao Shen, Yi Mao, Xiaoxia Du, Jun Ma

**Affiliations:** ^1^Department of Developmental and Behavioral Pediatrics, Shanghai Children's Medical Center, School of Medicine, Shanghai Jiao Tong University, Shanghai, China; ^2^School of Biomedical Engineering, Shanghai Jiao Tong University, Shanghai, China; ^3^College of Medical Imaging, Shanghai University of Medicine and Health Sciences, Shanghai, China; ^4^Department of Nephrology, Shanghai Children's Medical Center, School of Medicine, Shanghai Jiao Tong University, Shanghai, China; ^5^School of Psychology, Shanghai University of Sport, Shanghai, China

**Keywords:** primary nocturnal enuresis, resting-state functional connectivity, children, hippocampus, subregion

## Abstract

**Objective:**

Previous neuroimaging studies have shown abnormal brain-bladder control network in children with primary nocturnal enuresis (PNE). The hippocampus, which has long been considered to be an important nerve center for memory and emotion, has also been confirmed to be activating during micturition in several human imaging studies. However, few studies have explored hippocampus-related functional networks of PNE in children. In this study, the whole resting-state functional connectivity (RSFC) of hippocampus was investigated in children with PNE.

**Methods:**

Functional magnetic resonance imaging data of 30 children with PNE and 29 matched healthy controls (HCs) were analyzed in our study. We used the seed-based RSFC method to evaluate the functional connectivity of hippocampal subregions defined according to the Human Brainnetome Atlas. Correlation analyses were also processed to investigate their relationship with disease duration time, bed-wetting frequency, and bladder volume.

**Results:**

Compared with HCs, children with PNE showed abnormal RSFC of the left rostral hippocampus (rHipp) with right fusiform gyrus, right Rolandic operculum, left inferior parietal lobule, and right precentral gyrus, respectively. Moreover, decreased RSFC of the left caudal hippocampus (cHipp) with right fusiform gyrus and right supplementary motor area was discovered in the PNE group. There were no significant results in the right rHipp and cHipp seeds after multiple comparison corrections. In addition, disease duration time was negatively correlated with RSFC of the left rHipp with right Rolandic operculum (*r* = −0.386, *p* = 0.035, uncorrected) and the left cHipp with right fusiform gyrus (*r* = −0.483, *p* = 0.007, uncorrected) in the PNE group, respectively. In the Receiver Operating Characteristic (ROC) analysis, all the above results of RSFC achieved significant performance.

**Conclusions:**

To our knowledge, this is the first attempt to examine the RSFC patterns of hippocampal subregions in children with PNE. These findings indicated that children with PNE have potential dysfunctions in the limbic network, sensorimotor network, default mode network, and frontoparietal network. These networks may become less efficient with disease duration time, inducing impairments in brain-bladder control, cognition, memory, and emotion. Further prospective research with dynamic observation of brain imaging, bladder function, cognition, memory, and emotion is warranted.

## Introduction

Primary nocturnal enuresis (PNE) is the most common form of elimination disorder in childhood characterized by symptoms of intermittent incontinence during sleep without a previous dry period of more than 6 months (1, 2). Approximately 5–10% of 7-year-old children wet at night and 0.5–1% of adults still suffer from this condition, although the spontaneous remission rate is about 15% per year (3). The persistence of bed-wetting increases children's risk for physical and mental problems, such as chronically disturbed sleep, chronic lower self-esteem, and various psychiatric conditions (3, 4). Nevertheless, some children with PNE remain resistant to all clinically available treatments, largely because the pathogenesis of PNE is still unclear.

It is generally believed that nocturnal polyuria, arousal dysfunction, and abnormal nocturnal bladder function are three main factors implicated in the pathogenesis of PNE (4–6), and maturational delays of the central nervous system have been considered to be related to the pathogenesis of PNE (7). Over the past few decades, magnetic resonance imaging (MRI) technology, especially functional MRI (fMRI), has been validated as an efficient, non-invasive, and promising approach to investigating the neural mechanisms of bladder control in healthy and pathological participants (8). In recent years, brain MRI studies have shown that PNE is strongly linked to alterations in brain structure and function (7). Our previous resting-state fMRI study has found that spontaneous activity abnormalities of the prefrontal cortex (PFC) and midbrain are likely linked to micturition control impairment in children with PNE (9). Additionally, we have also found that structural changes in PFC and precuneus may be linked to micturition and sleep problems of PNE in children (10). The above brain regions are mainly involved in the neural circuits within a micturition control model presented by Griffiths (11). Apart from dysfunction in the bladder control during sleep, an inability to wake up from the sense of full bladder is another common complaint among parents of children suffering from bed-wetting. The insular activity is strongly associated with bladder sensation. One fMRI study on the control of low urinary tract in healthy subjects suggested that the activity of right anterior insular enhanced during “attempted micturition” in the full-bladder condition (12). Notably, hippocampus has been revealed as an integrated component implicated in interoception (13), such as visceral sensations. As a main part of the human limbic system, hippocampus, an elongated structure (14), has long been regarded as a major center for memory, cognition, and emotion (15). Yet, there could be more function in hippocampus. For example, hippocampal activation during micturition has been confirmed in human and animal imaging studies (16–18). A previous study on the neural network controlling rat's bladder, hippocampus has been labeled (19). Moreover, children with PNE have shown potential impairments in working memory (20, 21), response inhibition (22), attention (23), and emotional response (24) during various fMRI studies, which might be related to hippocampal activities. Taken together, PNE-related symptoms and behaviors may be influenced by interactions between hippocampus and other cortical regions. Along its longitudinal axis, hippocampus varies in structure, function, and connectivity. The anterior hippocampus is primarily responsible for emotion, whereas the posterior hippocampus is implicated in memory and cognition (25). Previous studies have shown abnormal RSFC patterns of the hippocampus in a great number of psychiatric diseases, such as depression (26, 27), schizophrenia (28, 29), and posttraumatic stress disorder (30). Nevertheless, the RSFC patterns of the hippocampus functionally linked to other brain areas in children with PNE have not been investigated so far.

Given the potential roles of hippocampus in bladder sensation, micturition, bladder control, PNE-related cognitive and emotional deficits, we aimed to explore the RSFC patterns of the hippocampus in children with PNE using a seed-based RSFC method in this work. We mainly hypothesized that there are anomaly RSFC patterns of hippocampal subregions with other cortical regions compared with healthy controls (HCs). The second analysis was to investigate the association between altered RSFC patterns of hippocampal subregions and clinical characteristics in children with PNE, and the predictive value of RSFC patterns of hippocampal subregions with other cortical regions, gaining access to key information that may have clinical implications.

## Materials and methods

### Participants

Thirty-three children with PNE were enrolled in the outpatient clinic of Shanghai Children's Medical Center, and 33 HCs matched for age and gender were recruited by advertisement. All patients had bed-wetting, with one or more episodes per month for at least 3 months, and were diagnosed by senior pediatricians following the International Children's Continence Society (ICCS) criteria (1). Their urine tests were normal without glucosuria and leukocytes. Ultrasound examination of their urinary systems uncovered no organic problems in the kidney, urinary tract, and bladder. Moreover, the bladder volume was acquired by the ultrasound when patients had a strong desire to void. We also collected information on the age, gender, disease duration time, and bed-wetting frequency by a questionnaire in which a detailed clinical history was recorded. HCs were no enuresis and could wake up in response to the sensation of full bladder during sleep since they were 5 years old. The clinical features of participants were presented in Table 1.

**Table 1:** Demographic and clinical data for PNE and HC children.

**Baseline characteristic**	**PNE group (*n* = 30)**	**HC group (*n* = 29)**	***P*** **-value**
Age, years, median (IQR)	8.5 (7–10)	8 (7–10)	0.969^a^
Gender, *n* (%)			0.358^b^
Male	14 (46.7)	17 (58.6)	
Female	16 (53.3)	12 (41.4)	
Handedness, *n* (%)			NA
Right	30 (100.0)	29 (100.0)	
Left	0 (0.0)	0 (0.0)	
Mean FD, mean ± SD	0.15 ± 0.05	0.19 ± 0.10	0.063^c^
Duration time, years, median (IQR)	3.5 (2–5)	NA	NA
Bed-wetting frequency, per week, median (IQR)	4 (1.75–6.25)	NA	NA
Bladder volume, ml, mean ± SD	177.03 ± 75.47	NA	NA

*PNE, primary nocturnal enuresis; HC, healthy control; IQR, interquartile range; FD, framewise displacement; SD, standard deviation; NA, not applicable;*

a *Mann-Whitney test;*

b *Chi-squared test;*

c *Two-sample t-test*.

The inclusion criteria of all participants were: 5–18 years; right-handedness; with an IQ above 75 (Wechsler Intelligence Scale for Children-Revised); a clinical assessment and diagnosis by senior developmental and behavioral pediatricians; without an organic history causing bed-wetting (e.g., diabetes mellitus, epilepsy, urinary infection); without any history of other psychiatric or neurological diseases (e.g., intellectual disability, attention-deficit/hyperactive disorder, autism spectrum disorder, cerebral palsy); without receiving any treatments or drugs about anti-enuresis before MRI scanning. And the exclusion criteria of all participants were: with any daytime lower urinary tract symptoms; left-handedness; with an IQ below 75; contraindications for MRI; with obvious head movement (translation > 2 mm, rotation > 2°) on these brain images; receiving any other antipsychotics.

Ethical approval was obtained from the IRB of Shanghai Children's Medical Center, School of Medicine, Shanghai Jiao Tong University (No: SCMC-201014). This study was conducted under the Declaration of Helsinki. All guardians and their children provided written informed consent before study enrollment.

### MRI data acquisition

The structural and functional MRI data were acquired on the 3.0 T MR imaging system (Prisma, Siemens, Germany) at Shanghai Key Laboratory of Magnetic Resonance (East China Normal University, Shanghai, China). fMRI sequence parameters were as follows: volume number = 240, acquisition matrix = 64 × 64, repetition time (TR)/echo time (TE) = 2,000/30 ms, voxel size = 3.5 × 3.5 × 3.5 mm^3^, acquisition time = 486 s, flip angle = 90°, field of view (FOV) = 224 × 224 mm^2^, slice number = 33. All children were told to stay awake and still, keeping their eyes closed during scanning. We also collected high-resolution T1-weighted images from all children, sequence parameters were set as follows: acquisition matrix = 256 × 256, inversion time = 1,100 ms, TR/TE = 2,530/2.98 ms, flip angle = 7°, FOV = 256 × 256 mm^2^, voxel size = 1 × 1 × 1 mm^3^, 192 slices (scan time of 361 s).

### Data preprocessing

All MRI data were preprocessed in the MATLAB 2014a (The MathWorks, Inc., Natick, Massachusetts, USA) using RESTplus version 1.24 (31), a toolkit based on SPM12 (http://www.fil.ion.ucl.ac.uk/spm/; Wellcome Trust Centre for Neuroimaging, University College London, UK). Preprocessing included these following steps: (1) removing the first 10 time points of each rs-fMRI data for participants' acclimatization and signal's stability; (2) slice-timing to correct the remaining time points; (3) realignment; (4) normalizing to Montreal Neurological Institute (MNI) template as well as resampling into a new 3 × 3 × 3 mm^3^ voxel size by using T1 image unified segmentation; (5) smoothing with a Gaussian kernel (full-width-half-maximum, FWHM of 6 mm); (6) detrending; (7) regressing common covariates out, including Friston's 24 head motion parameters (32), white matter (WM), and cerebrospinal fluid (CSF) signals; (8) filtering (0.01–0.08 Hz). There were 3 children with PNE and 4 HCs excluded for excessive head motion (translation > 2 mm, rotation > 2°). Thus, 30 children with PNE and 29 HCs were included in our final analyses.

### Resting-state functional connectivity analysis

**Figure 1:**
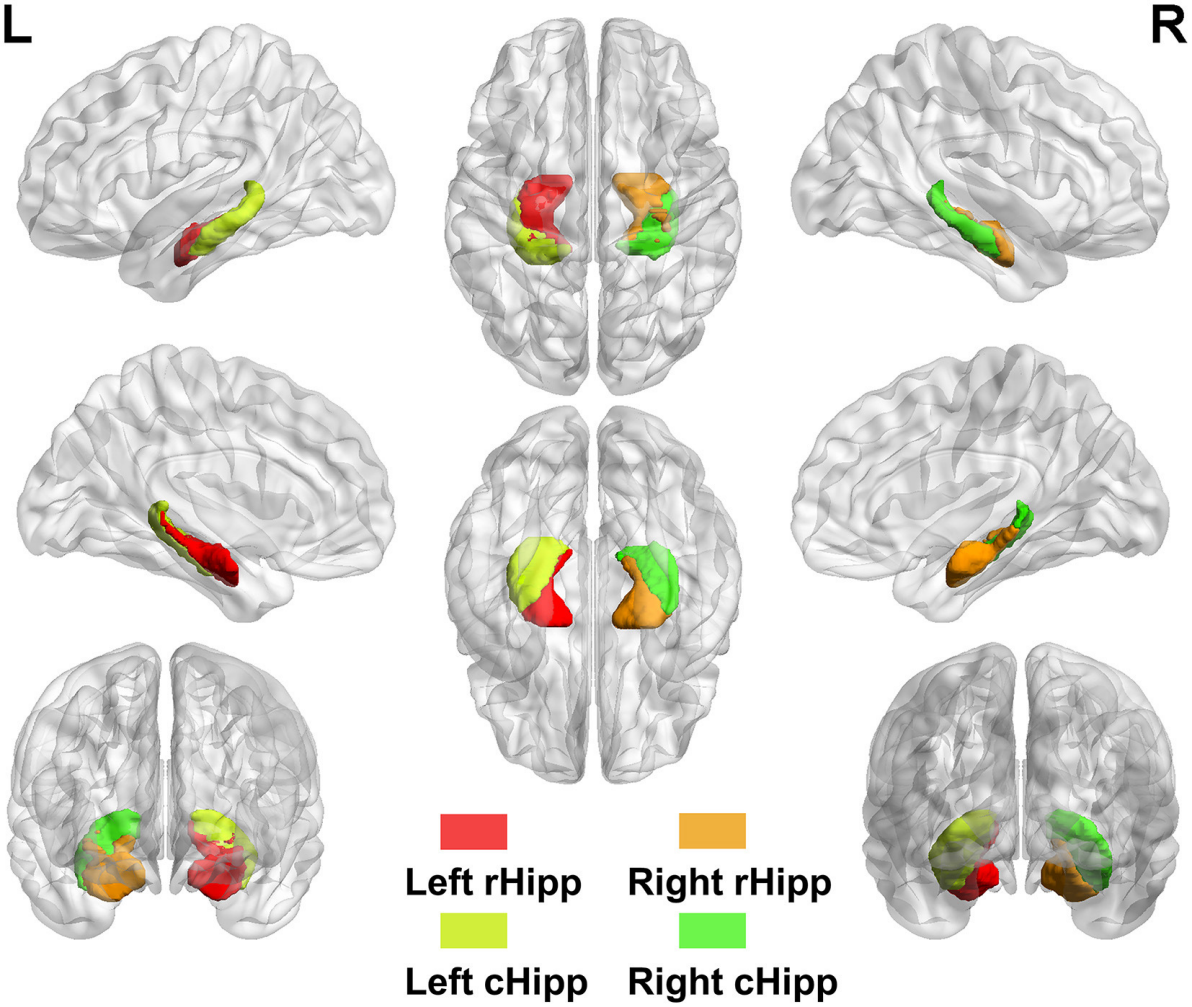
The anatomical location of hippocampal subregions. Hippocampal subregions based on the Human Brainnetome Atlas, including left rostral hippocampus (rHipp), right rostral hippocampus (rHipp), left caudal hippocampus (cHipp), and right caudal hippocampus (cHipp).

Analyses of the seed-based RSFC were conducted using the RESTplus version 1.24 toolkit. The bilateral hippocampi were divided into four subregions based on the Human Brainnetome Atlas (33), including left rostral hippocampus (rHipp), right rostral hippocampus (rHipp), left caudal hippocampus (cHipp), and right caudal hippocampus (cHipp) (Figure 1). First, we selected the left rHipp, right rHipp, left cHipp, and right cHipp as regions of interest (ROIs) for analyses. Then, the average time series of each hippocampal subregion seed was calculated in each subject to generate correlation maps by voxel-wise correlation coefficients, respectively. Finally, by using Fisher's r-to-z transformation, we converted these correlation coefficients into z-values to improve normality.

### Receiver operating characteristic analysis

The significantly altered RSFC z-values of hippocampal subregion seeds were extracted and used for the Receiver Operating Characteristic (ROC) analysis using MedCalc for Windows, version 18.2.1 (MedCalc Software, Ostend, Belgium). As a frequently used summary measure of the ROC curve, the maximum Youden index (sensitivity + specificity – 1) (34) and the corresponding sensitivity, specificity, 95% confidence intervals (CIs) for each altered brain region were computed.

### Statistical analysis

We conducted statistical analyses of RSFC using the RESTplus version 1.24 toolkit. First, group comparisons on the RSFC z-values derived from each hippocampal subregion seed between PNE and HCs groups were carried out *via* the two-sample *t*-test. The age and gender of each participant were set as covariates during the between-group comparisons in the RESTplus software to reduce their potential confounding effects on the results. Second, we utilized multiple comparison corrections, namely Gaussian Random Field (GRF) corrections, for RSFC results of all hippocampal subregion seeds (GRF correction, single-voxel *P* < 0.001 as well as cluster-level *P* < 0.05). Third, the abnormal RSFC z-values based on each hippocampal subregion seed after GRF correction were extracted. Then, we performed Person's correlation analysis between clinical characteristics (e.g., disease duration time, bed-wetting frequency, and bladder volume) and the above aberrant RSFC z-values in the PNE group.

## Results

### Demographic and clinical characteristics

Overall, 59 right-handed participants, 30 children with PNE [8.5 (7–10) years] and 29 HCs without enuresis [8 (7–10) years], were included in our final analyses. There were no differences in age, gender, and mean FD (35) between the groups (Table 1).

### Group comparisons in the RSFC of hippocampal subregions

#### rHipp

**Figure 2:**
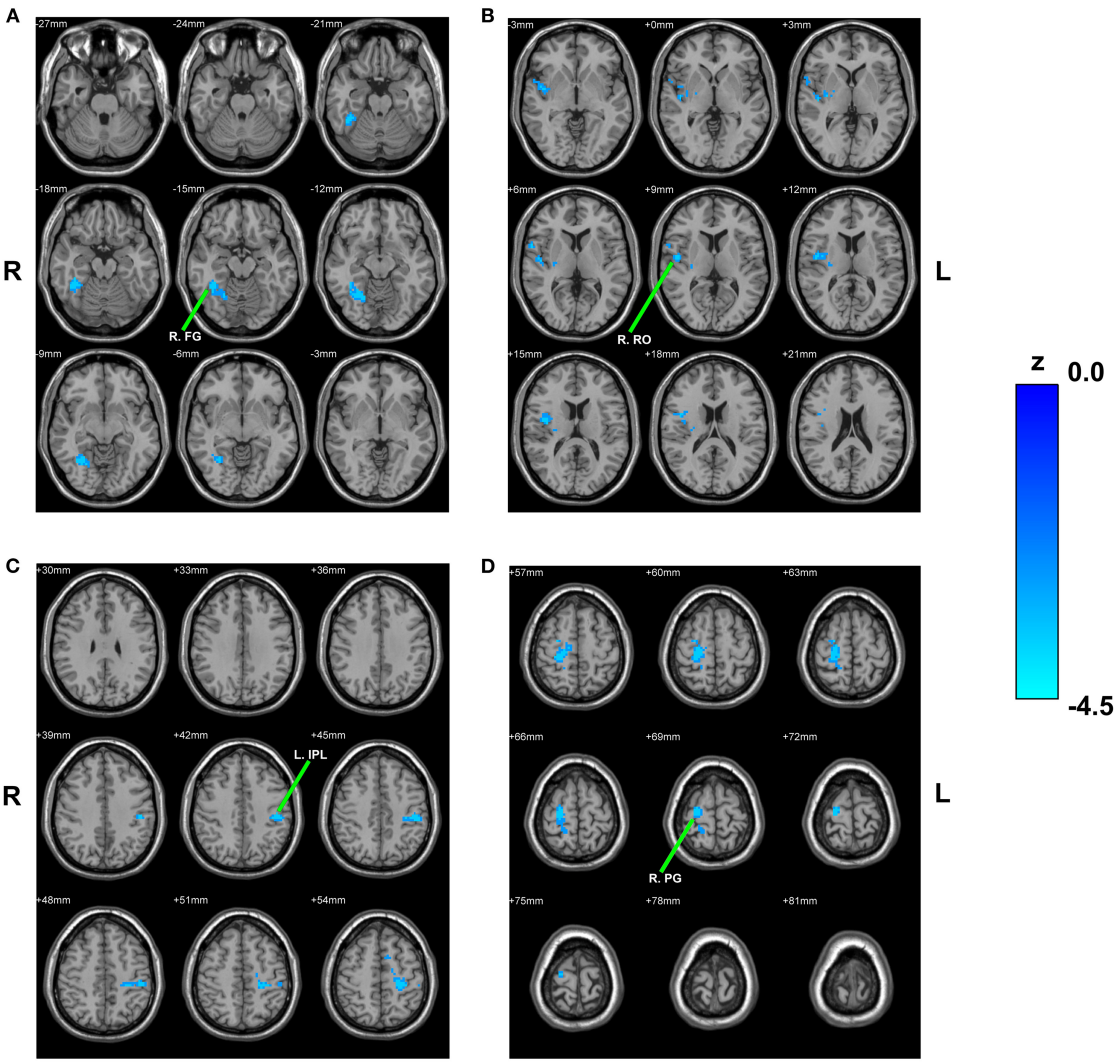
Between-group comparison based on the left rHipp. The reduced RSFC of the left rHipp with right fusiform gyrus **(A)**, right Rolandic operculum **(B)**, left inferior parietal lobule **(C)**, and right precentral gyrus **(D)** in children with PNE, respectively. rHipp, rostral hippocampus; PNE, primary nocturnal enuresis; R. FG, right fusiform gyrus; R. RO, right Rolandic operculum; L. IPL, left inferior parietal lobule; R. PG, right precentral gyrus; color bar, z value.

Compared with HCs, children with PNE showed decreased RSFC of the left rHipp with right fusiform gyrus, right Rolandic operculum, left inferior parietal lobule (IPL), and right precentral gyrus, respectively (Table 2; Figure 2). However, there were no significant RSFC results based on the right rHipp seed.

**Table 2:** Brain regions presenting significant differences in functional connectivity with left hippocampus between PNE and HC children (GRF corrected for single-voxel *P* < 0.001 and cluster-level *P* < 0.05).

**Brain regions**	**BA**	**Cluster size**	**MNI coordinates**			**Peak Z-value**
			**X**	**Y**	**Z**	
**Left rHipp seed**	NA	165	−22	−14	−19	NA
Right fusiform gyrus	NA	147	39	−45	−15	−4.2595
Right Rolandic operculum	NA	163	48	−9	9	−3.9642
Left inferior parietal lobule	NA	199	−45	−27	42	−4.2606
Right precentral gyrus	6	297	21	−18	69	−4.1855
**Left cHipp seed**	NA	172	−28	−30	−10	NA
Right fusiform gyrus	19	170	24	−60	−12	−4.4981
Right supplementary motor area	24	168	9	−6	48	−4.1969

*BA, Brodmann's area; PNE, primary nocturnal enuresis; HC, healthy control; MNI, Montreal Neurological Institute; NA, not applicable; rHipp, rostral hippocampus; cHipp, caudal hippocampus; hippocampal subregions seeds were transformed into MNI coordinates according to the Human Brainnetome Atlas*.

#### cHipp

**Figure 3:**
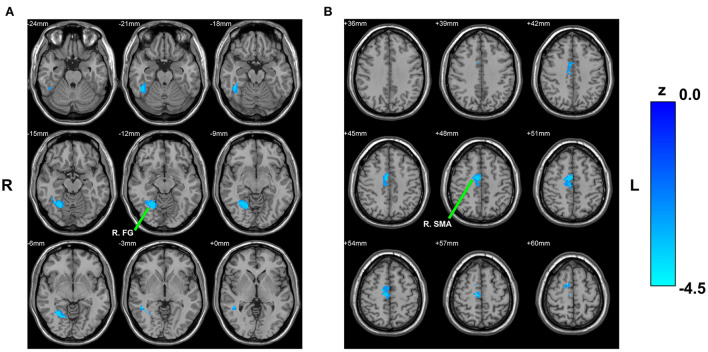
Between-group comparison based on the left cHipp. The decreased RSFC of the left cHipp with right fusiform gyrus **(A)** and right supplementary motor area **(B)** in children with PNE, respectively. cHipp, caudal hippocampus; PNE, primary nocturnal enuresis; R. FG, right fusiform gyrus; R. SMA, right supplementary motor area; color bar, z value.

Reduced RSFC of the left cHipp with right fusiform gyrus and right supplementary motor area was also discovered in the PNE group relative to the HCs group (Table 2; Figure 3). Nevertheless, we found no differences in the RSFC of the right cHipp seed between the groups.

### ROC curves results

**Figure 4:**
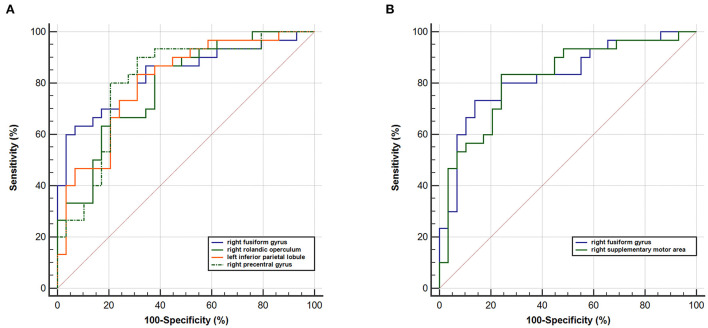
ROC curve analysis. ROC curve for altered RSFC patterns based on the left **(A)** rHipp and **(B)** cHipp to distinguish children with PNE from HCs. rHipp, rostral hippocampus; cHipp, caudal hippocampus; PNE, primary nocturnal enuresis; HCs, healthy controls.

When taking into account the area under the curve (AUC) and 95% CIs, it was determined that the RSFC z-value between the left rHipp and right fusiform gyrus exhibited the most accurate classification. For AUC, the altered brain regions all reached significant levels of *p* < 0.0001 (Table 3; Figure 4).

**Table 3:** The ROC analysis for altered brain regions that distinguish PNE children from HC children.

**Brain regions**	**SEN**	**SPE**	**AUC**	**95% CI**
**Left rostral hippocampus**				
zFC_right fusiform gyrus	60.00%	96.55%	0.832	0.712–0.917
zFC_right Rolandic operculum	86.67%	62.07%	0.790	0.664–0.885
zFC_left inferior parietal lobe	83.33%	68.97%	0.805	0.681–0.896
zFC_right precentral gyrus	80.00%	79.31%	0.808	0.685–0.899
**Left caudal hippocampus**				
zFC_right fusiform gyrus	73.33%	86.21%	0.825	0.704–0.912
zFC_right supplementary motor area	83.33%	75.86%	0.821	0.699–0.908

*SEN, sensitivity; SPE, specificity; AUC, area under the ROC curve; CI, confidence interval; PNE, primary nocturnal enuresis; HC, healthy control; zFC, functional connectivity z values*.

### Relationship between clinical characteristics and RSFC

**Figure 5:**
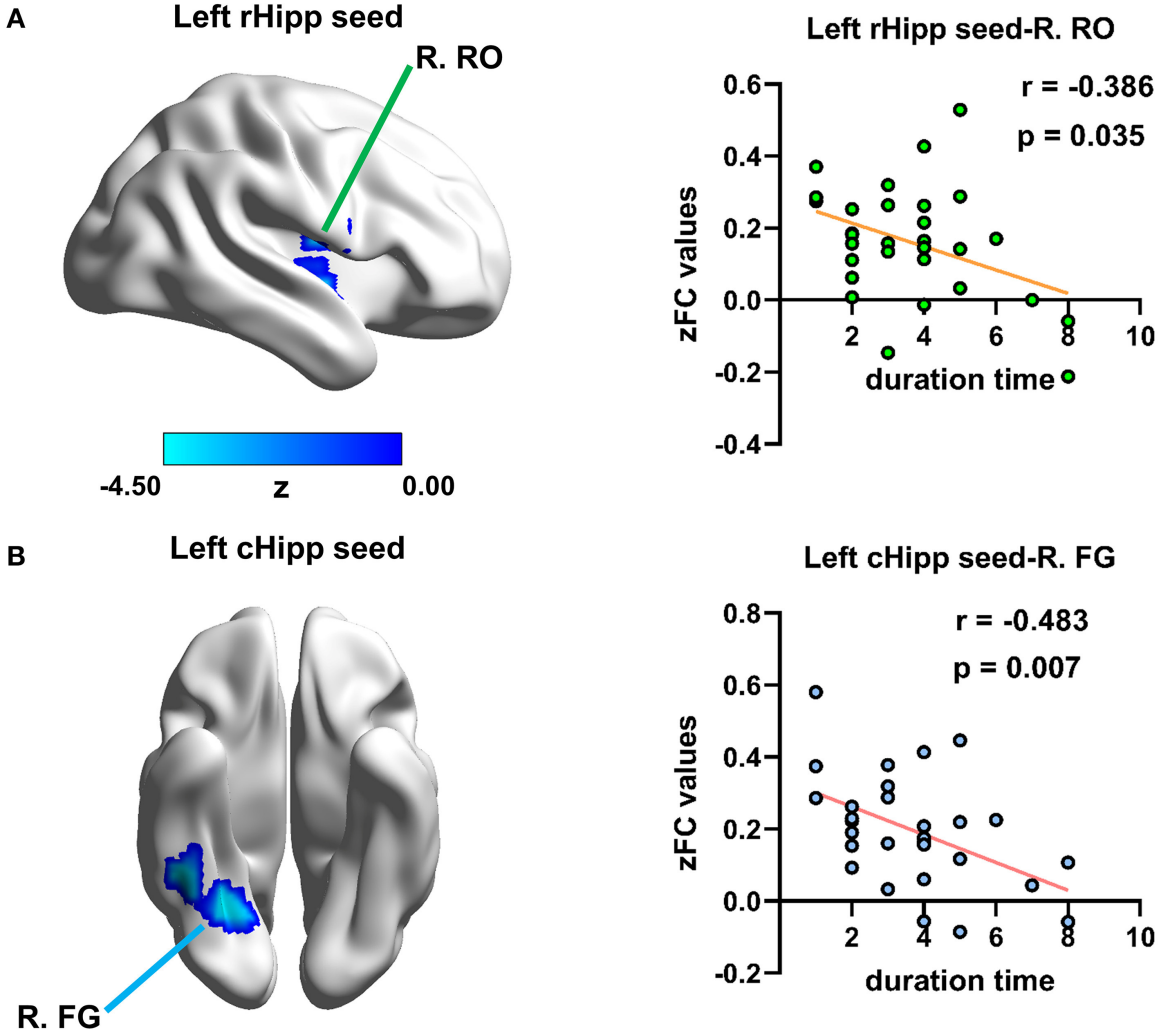
Relationship between disease duration time and altered RSFC values in children with PNE. Disease duration time was negatively correlated with RSFC of **(A)** the left rHipp with right Rolandic operculum and **(B)** the left cHipp with right fusiform gyrus in the PNE group, respectively. rHipp, rostral hippocampus; cHipp, caudal hippocampus; PNE, primary nocturnal enuresis; R. RO, right Rolandic operculum; R. FG, right fusiform gyrus; color bar, z value.

Disease duration time was negatively correlated with RSFC of the left rHipp with right Rolandic operculum (*r* = −0.386, *p* = 0.035, uncorrected) and the left cHipp with right fusiform gyrus (*r* = −0.483, *p* = 0.007, uncorrected) in the PNE group, respectively (Figure 5), yet there were no significant relationships between bet-wetting frequency or bladder volume and RSFC z-values of significantly altered brain clusters.

## Discussion

Compared with HCs, we primarily found that children with PNE showed abnormal RSFC of the left rHipp with right fusiform gyrus, right Rolandic operculum, left IPL, and right precentral gyrus, respectively. The reduced RSFC of the left cHipp with right fusiform gyrus and right supplementary motor area was also discovered in children with PNE. However, there were no statistically significant clusters based on the right rHipp and cHipp seeds. Furthermore, disease duration time was negatively correlated with RSFC for the left rHipp with right Rolandic operculum in the PNE group. RSFC for the left cHipp with right fusiform gyrus was also negatively associated with disease duration time in the PNE group. In the ROC analysis, all the above results of RSFC were significant. Based on these findings, children with PNE may have potential dysfunctions in the limbic network, sensorimotor network, default mode network (DMN), and frontoparietal network, which may become less efficient with disease duration. The above networks are involved in bladder sensation, low urinary tract control, cognition, memory, and emotion. Therefore, further prospective research that ongoing observation of brain imaging, bladder function, cognition, memory, and emotion is warranted.

One of our main results was that decreased RSFC of the left rHipp with right fusiform gyrus was uncovered in children with PNE relative to HCs. The rHipp, namely the anterior hippocampus, contributes to episodic memory, imagination, and visual scene perception, with widespread connectivity (15). The fusiform gyrus is considered to be implicated in the processing of high-order visual functions, such as face perception, object recognition, and reading (36). A categorical n-back task fMRI study indicated that there was a lower percentage of correct responses and longer mean reaction time to correct response in children with PNE, possibly associated with dysfunction in the left cerebella (21). Although there was no significant difference in the hippocampus and fusiform gyrus between-group comparison, the fusiform gyrus was activated in both PNE children and controls in this study (21). In another fMRI research, the hippocampus and fusiform gyrus have been proven to be engaged in different kinds of categorization learning (37). Therefore, the interaction of the hippocampus and fusiform gyrus might play a vital role in the cognitive impairments of PNE children.

Our results also exhibited reduced RSFC between the left rHipp and some sensorimotor areas, including the right Rolandic operculum and right precentral gyrus. Integrated exteroceptive-interoceptive signals are processed by the Rolandic operculum for bodily self-awareness and interoceptive awareness (38, 39). The hippocampus also plays an important role in interoception (13). It is well known that children with PNE have difficulty waking up to bladder signals during sleep. Therefore, the abnormal RSFC between the left rHipp and Rolandic operculum might be implicated in micturition desire-awakening in children with PNE. Post-stroke patients can experience intense psychological symptoms following stroke (e.g., apathy, depression, anxiety, and stress) if the right Rolandic operculum is damaged (40). As mentioned before, the rHipp relates to stress, emotion, and affect (25). Bed-wetting tends to be rated as one of the most stressful life events for children (4). Hence stress coupled with rHipp change might present in children with PNE. Moreover, we found that bed-wetting duration time was negatively correlated with RSFC of the left rHipp with right Rolandic operculum in the PNE group. In clinical practice, children with persistent bed-wetting often have secondary psychological problems, such as low self-esteem, anxiety and depression, though the problems may be mild.

On the other hand, the precentral gyrus is where the primary motor cortex is located (41). Our previous fMRI study (42) found that the reduced RSFC between the precentral gyrus and thalamus in children with PNE might be related to arousal dysfunction. The primary motor cortex is not only a structure for controlling motor movements but also a dynamic substrate that may also contribute to motor learning and cognitive processes (43). Children with PNE displayed a slower motor performance than controls, especially repetitive hand and finger movements, suggesting a possible maturation deficit in the motor cortex circuitry (44). Furthermore, pathologic performance on visuomotor integration abilities was more prevalent in PNE children, despite not experiencing impairment in visual or motor tasks (45). In a word, these findings demonstrated that the abnormal RSFC between the left rHipp and some sensorimotor areas may be associated with mild neuromotor development delay in children with PNE.

As an integral part of the DMN, the left IPL plays an essential role in a variety of higher cognitive functions (46). The well-known DMN, which is active at rest condition and deactivated when external attention is required, plays an important role in maintaining continence together with the salience network (11). In our results, the decreased RSFC between the left rHipp and left IPL was also discovered in children with PNE. As mentioned before, the hippocampal activation is observed during micturition in human and animal (16–18). Taken together, we assumed that the interplay of DMN and limbic network through left IPL might be involved in micturition problem in children with PNE.

Another important finding in the present work showed that children with PNE had reduced RSFC of the left cHipp with the right fusiform gyrus and right supplementary motor area. The cHipp, namely the posterior hippocampus, performs primarily cognitive functions (25). As mentioned before, the fusiform gyrus is implicated in the processing of high-level visual functions (36). Evidence from some fMRI studies supported the hypothesis that episodic memory performance is linked to the anterior hippocampus, while spatial memory performance is associated with the posterior hippocampus (47, 48). In addition, we found that bed-wetting duration time was negatively correlated with RSFC for the left cHipp with right fusiform gyrus in the PNE group. Thus, we inferred that the left cHipp might play a different role in the cognitive deficit of PNE children. In a prospective study, short-term memory improved after desmopressin treatment in children with PNE (49), indicating the action of desmopressin treatment on the central nervous system, not simply on the kidney. The central vasopressin receptors are abundantly expressed in the hippocampus (50). These findings may provide additional evidence to confirm that the hippocampus is involved in cognitive deficit in children with PNE. For another thing, the supplementary motor area is mainly related to motor-related functions as well as speech and language processing (51). Interestingly, bed-wetting is associated with neuromotor and language development (45, 52).

In this study, there were no significant results based on the right rHipp and cHipp seeds after multiple comparison corrections. This might result from left-right hemispheric differences of the hippocampus. A meta-analysis study found intra- and interhemispheric differences in anterior and posterior functional and structural connectivity, between the right and left hippocampi (53). However, further study is needed to explore the varying functions of the right and left hippocampi. Based on our results, the abnormal brain areas are not simply related to micturition control in children with PNE but are also associated with cognition, emotion, neuromotor, and language development. Thus, we inferred that PNE might be related to brain cognitive and emotional change which has also been proved by many other studies. Sleep is strongly associated with vigilant attention (54), memory (55), emotion regulation (56), and academic performance (57). It is well established that sleep problems, such as sleep fragmentation and daytime sleepiness, are more common in children with PNE (58). Children with PNE often suffer from poor sleep quality (59). In many cases, sleep on the one hand, and developmental delay on the other, have led to mild cognitive, memory, and emotion disturbance in children with PNE. In this study, we indicated that bed-wetting duration time was negatively correlated with RSFC for the left rHipp with right Rolandic operculum and the left cHipp with right fusiform gyrus in the PNE group, respectively. Based on previous literature, persistent bed-wetting might harm children's brain function and corresponding behavior. The present study offered more evidence for the link between PNE and brain function, the brain function might get worse with PNE duration time increased. In the ROC analysis, results of RSFC based on hippocampal subregions obtained significant performance, suggesting that these RSFC values might be used as potential neuroimaging marks to distinguish children with PNE from HCs, or potential predictive factors of therapeutic effectiveness. Further studies are warranted to combine these crucial indices to achieve a higher prediction precision.

The strengths of our study include two sides. First, it is novel to explore the RSFC patterns of hippocampus in children with PNE. Second, merely focusing on some proven brain regions may limit our knowledge of the brain basis of PNE, our study provided a new insight into PNE-related deficits. The growing knowledge may continue to expand the pathological model of PNE. Nevertheless, there are some limitations in this study which need to be addressed in the future. First, the relatively small sample size might reduce the statistical power of our results and therefore more participants need to be included for validating our findings in later research. Second, the collected clinical data were relatively few, especially in detailed behavior assessment, that may limit our comprehensive analysis on relationship between brain and behavior in children with PNE. Third, we only selected four parts of hippocampus as seeds, but this is not a very delicate anatomical segmentation, and as such it could have been missed out some important information. The more refined parceling methods should be considered in future studies. Fourth, confounders such as age, gender, emotion status, and bladder volume were not taken into account for the correlation analysis. Fifth, we only explored a simple correlation between brain and clinical information in children with PNE, but the causality could not be determined.

## Conclusion

In this study, we examined the RSFC patterns of hippocampal subregions in children with PNE, which is the first attempt to our knowledge. The findings indicated that children with PNE may have potential dysfunctions in the limbic network, sensorimotor network, DMN, and frontoparietal network, which may become less efficient when bed-wetting persists without timely treatment, inducing impairments in brain-bladder control, cognition, memory, and emotion. Further prospective research with ongoing observation of brain imaging, bladder function, cognition, memory, and emotion is warranted.

## Data availability statement

The raw data supporting the conclusions of this article will be made available by the authors, without undue reservation.

## Ethics statement

The studies involving human participants were reviewed and approved by the IRB of Shanghai Children's Medical Center, School of Medicine, Shanghai Jiao Tong University. Written informed consent to participate in this study was provided by the participants' legal guardian/next of kin.

## Author contributions

JM, SZ, LZ, and XD co-designed this study. SZ and LZ completed all data analysis relevant to this work and drafted the initial manuscript. MW, JS, and YM acquired the data. XD and JM revised the manuscript. All authors reviewed and approved the final manuscript. All authors contributed to the article and approved the submitted version.

## Funding

This work was supported by grants from the Science and Technology Commission of Shanghai Municipality (20ZR1434700) and the National Natural Science Foundation of China (81901720 and 62001292).

## Conflict of interest

The authors declare that the research was conducted in the absence of any commercial or financial relationships that could be construed as a potential conflict of interest.

## Publisher's note

All claims expressed in this article are solely those of the authors and do not necessarily represent those of their affiliated organizations, or those of the publisher, the editors and the reviewers. Any product that may be evaluated in this article, or claim that may be made by its manufacturer, is not guaranteed or endorsed by the publisher.
